# Effects of Chronic Paroxetine Exposure and Irisin Treatment on Sexual Motivation, Reproductive Endocrine Profile, and Vaginal Histological and Immune-Inflammatory Outcomes in Adult Female Rats

**DOI:** 10.3390/biology15191767

**Published:** 2026-10-04

**Authors:** Ahmet Yardimci, Tugrul Ertugrul, Nazife Ulker Ertugrul, Sinan Canpolat

**Affiliations:** 1 Department of Physiology, Faculty of Medicine, Firat University, 23119 Elazig, Turkey; 2 Department of Histology and Embryology, Faculty of Veterinary Medicine, Ondokuz Mayis University, 55200 Samsun, Turkey; 3 Department of Physiology, Faculty of Medicine, Samsun University, 55080 Samsun, Turkey; 4 Department of Physiology, Faculty of Medicine, Dokuz Eylul University, 35220 Izmir, Turkey

**Keywords:** selective serotonin reuptake inhibitors, paroxetine, irisin, sexual motivation, active investigation behaviors, toll-like receptor 4, nuclear factor-kappa B, fibroblast, plasma cell

## Abstract

Selective serotonin reuptake inhibitors (SSRIs), particularly paroxetine, are frequently associated with sexual dysfunction, yet their effects on female reproductive endocrine regulation and the local vaginal tissue environment remain incompletely characterized. Irisin is an exercise-responsive adipomyokine with reported reproductive and immunomodulatory actions, but its influence during chronic paroxetine exposure is unknown. This study evaluated the behavioral, endocrine, histological, and immune-related effects of chronic paroxetine treatment and examined whether irisin changed responses. Forty regularly cycling female Sprague–Dawley rats were allocated to sham control, paroxetine, irisin, or paroxetine + irisin groups (*n* = 10/group). Paroxetine was administered orally at 20 mg/kg/day for 8 weeks, while irisin was delivered continuously by subcutaneous mini-osmotic pumps at 100 ng/kg/day during the final 4 weeks. Sexual incentive motivation and active investigation behaviors, serum luteinizing hormone (LH), follicle-stimulating hormone (FSH), testosterone, and anti-Müllerian hormone (AMH), vaginal fibroblast and plasma cell numbers, Toll-like receptor 4 (TLR4) and nuclear factor-kappa B (NF-κB) immunoreactivity, and periodic acid–Schiff (PAS) reactivity were evaluated. Paroxetine did not significantly affect sexual incentive motivation parameters or circulating reproductive hormone concentrations (*p* > 0.05), but both paroxetine-treated groups showed a lower duration of investigation directed toward the female and a higher male investigation preference ratio than the sham control (*p* < 0.05). Irisin alone reduced locomotor activity (*p* < 0.01). Paroxetine increased vaginal fibroblast counts (*p* = 0.0001) and NF-κB immunoreactivity (*p* = 0.003) compared with the sham control, whereas TLR4 immunoreactivity remained unchanged. Plasma cell counts were elevated in all treatment groups compared with the sham control (*p* < 0.0001). Irisin co-treatment reduced fibroblast counts relative to paroxetine group and reduced plasma cell counts relative to both single-treatment groups (all *p* < 0.001). Vaginal epithelial PAS reactivity was severe (+++) in sham control, weak (+) after paroxetine, and moderate (++) in both irisin-treated groups. Overall, chronic paroxetine exposure produced selective behavioral and local vaginal stromal, immune-inflammatory, and epithelial alterations without significant changes in the measured reproductive hormonal profile. Irisin influenced several vaginal tissue responses but did not change the paroxetine-associated alterations in active investigation behavior.

## Simple Summary

Selective serotonin reuptake inhibitors (SSRIs) such as paroxetine are widely used antidepressants, yet their effects on female reproductive function may extend beyond sexual behavior and endocrine regulation to local vaginal structural and immune-inflammatory changes in reproductive tissues. Irisin is an exercise-responsive adipomyokine implicated in female reproductive regulation and inflammatory signaling, prompting investigation of whether reproductive and vaginal tissue responses to chronic paroxetine exposure differ when irisin is co-administered. This study investigated whether chronic paroxetine exposure alters female sexual motivation, reproductive hormone levels, and vaginal stromal, immune, and epithelial responses, including fibroblast and plasma cell numbers, Toll-like receptor 4 (TLR4) and nuclear factor-kappa B (NF-κB) immunoreactivity, and PAS reactivity, and whether these responses differ in the presence of irisin. Female rats received paroxetine and/or irisin and were evaluated using behavioral, hormonal, histological, and immunohistochemical methods. Paroxetine altered active investigation behavior and produced marked changes in vaginal fibroblast and plasma cell numbers, NF-κB immunoreactivity, and apical PAS reactivity, whereas sexual incentive motivation, circulating reproductive hormones, and TLR4 immunoreactivity remained unchanged. Several vaginal tissue responses differed in the presence of irisin, whereas the paroxetine-associated behavioral alterations persisted. Overall, the findings show that chronic paroxetine exposure is associated with selective behavioral and local vaginal tissue changes, including immune-inflammatory alterations, without significant changes in circulating reproductive hormone levels, whereas irisin changed several vaginal tissue responses but did not change the behavioral alterations associated with paroxetine.

## 1. Introduction

### 1.1. Paroxetine and Sexual Motivation

Selective serotonin reuptake inhibitors (SSRIs) are one of the most frequently prescribed antidepressants for mental health disorders. These medications are highly effective in the treatment of depression and may improve general quality of life [1]. Moreover, the medical expenditure panel survey in 2013 showed that the percentage use of antidepressants, including SSRIs, in women was at double the rate of men (15.9% vs. 7.7%) [2]. Despite their well-established therapeutic efficacy, adverse effects associated with SSRI treatment may negatively affect treatment adherence. Sexual dysfunction is among the most frequently reported and disruptive adverse effects and commonly manifests in women as difficulties with sexual desire, arousal, and orgasm [1]. Although sexual dysfunction may occur with all SSRIs, paroxetine, which is widely used in clinical practice, has been associated with a higher risk than several other agents in this class [3,4]. Accordingly, paroxetine is widely used in experimental studies investigating SSRI-associated sexual dysfunction. Because sexual desire in women is conceptually related to the appetitive phase of sexual behavior in female rats, assessment of sexual incentive motivation in female rat models provides a useful translational approach to examining the motivational dimension of female sexual function [5,6]. In the sexual incentive motivation paradigm, the experimental female is simultaneously exposed to a male sexual stimulus and a female social stimulus in separate compartments, allowing motivational preference to be assessed from the time spent near each stimulus. The principal measures include the time spent near the male rat (TWM), the time spent near the female rat (TWF), total social time (TST; defined as the sum of TWM and TWF), and the male preference ratio (calculated as TWM/TST), while active investigation is quantified as the duration of investigation directed toward the male rat (TIM), the duration of investigation directed toward the female rat (TIF), total investigation time (TIT; TIM + TIF), and the male investigation preference ratio (TIM/TIT). Within this experimental framework, evidence regarding the effects of chronic paroxetine exposure remains limited and inconsistent. Chronic paroxetine administration has been reported to reduce sexual incentive motivation [7], whereas no significant alteration was observed in our recent study [8].

### 1.2. Paroxetine and Reproductive Endocrine Regulation

Beyond its effects on sexual function, paroxetine may also interfere with female reproductive endocrine regulation. Available preclinical findings on the reproductive endocrine effects of paroxetine remain limited and variable. Paroxetine administration has been reported to decrease serum follicle-stimulating hormone (FSH), luteinizing hormone (LH), progesterone, and estradiol levels, while increasing testosterone in female rats [9]. In our previous studies, paroxetine produced distinct endocrine alterations. Long-term paroxetine administration significantly increased serum estradiol levels without significantly affecting FSH, LH, or anti-Müllerian hormone (AMH) concentrations in female rats [10]. More recently, we demonstrated that a shorter-term paroxetine treatment significantly decreased serum AMH levels in female rats, although serum estradiol, progesterone, and testosterone levels remained unchanged [8]. Collectively, paroxetine can alter female reproductive endocrine homeostasis, although the direction and extent of these alterations may vary depending on treatment duration and other experimental conditions. Its effects on gonadotropins, ovarian steroids, and AMH further indicate a broader disturbance in reproductive hormonal regulation.

### 1.3. Paroxetine and Vaginal Tissue Responses

In addition to its effects on sexual motivation and reproductive endocrine regulation, paroxetine may also affect peripheral reproductive tissues. In this context, the vagina represents an essential target for determining whether paroxetine-associated alterations extend to the lower female genital tract. The vaginal wall plays a critical role in reproductive health by providing structural integrity, tissue elasticity, and immune protection [11]. It contains multiple cell populations, including epithelial cells, fibroblasts, smooth-muscle cells, and plasma cells [12]. Among these populations, fibroblasts and plasma cells are particularly relevant because they represent two distinct but essential aspects of the local vaginal tissue environment. Vaginal fibroblasts are major stromal cells involved in extracellular-matrix production and therefore contribute to the remodeling, structural homeostasis, and repair capacity of the vaginal wall [11]. In this context, dysregulation of fibroblast function has been implicated in several gynecological conditions, including pelvic organ prolapse, vaginal atrophy, and postoperative scarring, which may markedly compromise women’s quality of life [13,14,15]. Given this biological and clinical relevance, quantification of vaginal fibroblasts may provide an initial indication of whether paroxetine affects the stromal cellular compartment of the vagina.

Plasma cells, in turn, are essential effector cells of humoral immunity because they produce and secrete soluble immunoglobulins [16]. Plasma cells are abundant in the endocervix but comparatively scarce in the vagina, indicating the presence of local immunological microenvironments in the lower female genital tract [17]. Vaginal immunization has been shown to markedly increase vaginal plasma cell numbers and local antibody levels [18]. Together, vaginal plasma cells constitute an inducible, locally antibody-secreting population and may therefore serve as a useful cellular indicator of alterations in the local humoral compartment.

Local immune and inflammatory mechanisms may also contribute to responses in reproductive tissues [19]. In our previous studies, chronic paroxetine exposure was associated with increased inflammatory cell infiltration and vascular dilatation in the endometrial stroma, as well as an increased number of toluidine blue-positive uterine mast cells [10,20]. These findings suggest that paroxetine may influence the local immune environment of female reproductive tissues; however, its effects on immune-related signaling in vaginal tissue remain unknown. Given its central role in the initiation and regulation of innate inflammatory responses, the Toll-like receptor 4 (TLR4)-nuclear factor-kappa B (NF-κB) axis was selected as a key signaling pathway for investigation. Toll-like receptors (TLRs) are a family of transmembrane proteins and play an essential role in signal transduction. TLR4 is the cardinal sensor for detecting the pathogen-associated molecular patterns. TLR4, a member of the IL-1R/TLR superfamily, is essential for lipopolysaccharide-mediated signaling and acts in the host defense against Gram-negative bacteria [21]. TLR4 transduces signals through two principal adaptor-dependent pathways mediated by MyD88 and TRIF. The MyD88-dependent pathway predominantly activates NF-κB and mitogen-activated protein kinase signaling, thereby inducing the expression of inflammatory cytokine genes [22]. In female reproductive tissues, TLR4- and/or NF-κB-associated signaling has been implicated in epithelial barrier responses, stromal cell responses, cytokine secretion, and immune cell recruitment [23,24,25,26]. Accordingly, evaluation of TLR4 and NF-κB immunoreactivity in vaginal tissue may provide insight into whether paroxetine alters local innate immune-related signaling. However, the potential effects of paroxetine on the vaginal TLR4/NF-κB axis and the accompanying local immune response remain largely unexplored.

### 1.4. Irisin as a Potential Modulator

Irisin is an exercise-responsive myokine generated through the proteolytic cleavage of fibronectin type III domain-containing protein 5 (FNDC5) and released from skeletal muscle [27]. Our previous studies demonstrated that chronic irisin exposure can influence female reproductive endocrine regulation and the motivational component of sexual behavior, suggesting that irisin may act across both endocrine and behavioral dimensions of female reproductive function [28,29]. In addition to its endocrine effects, irisin exhibits immunomodulatory properties. It has been shown to suppress TLR4/MyD88/NF-κB-related signaling in response to lipopolysaccharide stimulation and to attenuate inflammatory alterations in experimental endometritis by modulating the AMPK/NF-κB pathway [30,31]. However, the effects of irisin on vaginal fibroblast and plasma cell populations have not yet been characterized.

### 1.5. Rationale and Aim of the Study

Our previous studies focused on paroxetine-related ovarian and reproductive endocrine alterations [8,10], uterine inflammatory and mast-cell responses [20], and the effects of irisin on reproductive endocrine regulation and sexual incentive motivation [28,29]. The present study extends our previous work to the lower female genital tract by examining vaginal stromal and immune cell populations, epithelial PAS reactivity, and TLR4/NF-κB immunoreactivity during chronic paroxetine exposure and assessing the potential modulatory effects of irisin. This approach allows us to determine whether paroxetine-associated reproductive alterations extend to the local cellular and innate immune-related environment of the vagina and whether irisin affects these responses.

The present study aimed to characterize the effects of chronic paroxetine exposure and irisin administration on sexual incentive motivation, reproductive endocrine regulation, and vaginal tissue responses in female rats. We hypothesized that chronic paroxetine exposure would be associated with behavioral, endocrine, stromal, immune-inflammatory, and epithelial alterations and that irisin administration could change these responses. Accordingly, sexual incentive motivation and circulating reproductive hormones were assessed alongside vaginal fibroblast and plasma cell numbers, TLR4 and NF-κB immunoreactivity, and vaginal epithelial PAS reactivity.

## 2. Materials and Methods

### 2.1. Sample Size Calculation

An a priori power analysis was performed using G*Power software (version 3.1.9.7, Heinrich-Heine-Universität Düsseldorf, Germany) for a one-way ANOVA involving four independent groups. An effect size of *f* = 0.60 was selected to represent a large expected effect according to Cohen’s conventions [32], with reference to a previous preclinical rat study using comparable one-way ANOVA design [33]. Based on an α level of 0.05, and a statistical power of 0.85, a total sample size of 40 animals was used, with 10 animals allocated to each experimental group.

### 2.2. Experimental Animals and Housing Conditions

Forty female Sprague–Dawley rats (approximately 2–3 months old, weighing 200–250 g) with verified regular estrous cycles were obtained from the Experimental Research Unit of Firat University (Elazig, Turkey). The animals were housed under standard laboratory conditions at a controlled temperature of 21 ± 1 °C and relative humidity of 55 ± 5%, with a 12 h light/12 h dark cycle (lights on at 07:00). Rats were housed in Plexiglas cages in groups of three or four and provided food and water *ad libitum*.

### 2.3. Estrous Cycle Monitoring

Estrous cyclicity was monitored by daily vaginal lavage for approximately 12–15 days before the initiation of the experimental procedures [34]. Only rats exhibiting at least two consecutive regular estrous cycles of 4–5 days each were considered to have normal cyclicity and were eligible for experiments [35]. Vaginal lavages were assessed by an experienced researcher, and the protocol was performed according to our previous study [29]. Following confirmation of regular estrous cyclicity, the animals were randomly allocated to four experimental groups (*n* = 10 per group)—sham control, paroxetine, irisin, and paroxetine + irisin—using simple randomization. The allocation sequence was generated using a computer-based random-number generator. One researcher remained aware of group allocation to coordinate treatment administration, whereas the other researchers responsible for behavioral assessments, ELISA measurements, histological and immunohistochemical evaluations, and statistical analyses were blinded to the experimental groups.

### 2.4. Paroxetine Administration and Assessment of Estrous-Cycle Regularity

Rats in the paroxetine and paroxetine + irisin groups received paroxetine at a dose of 20 mg/kg/day by oral gavage between 10:00 and 12:00 throughout the 8-week experimental period. Paroxetine tablets (Paxil, GlaxoSmithKline, Istanbul, Turkey) were finely ground, suspended in 0.2 mL of deionized water, and freshly prepared for each animal immediately before administration. During the same period, rats in the sham control group received an equivalent volume of deionized water (0.2 mL/day) by oral gavage. The irisin-only group did not receive daily vehicle gavage during the experimental period. The paroxetine dose was selected with reference to the chronic oral treatment regimen described by de Jong et al. (2005) [36]. The 8-week treatment duration, as well as drug preparation and administration, were based on our previous study, in which 20 mg/kg/day paroxetine markedly suppressed sexual activity in male rats [37].

Following the first 2 weeks of treatment, estrous cyclicity was monitored daily for 14 consecutive days using vaginal cytology, in accordance with established procedures for assessing estrous-cycle regularity in rats [34]. Because regular estrous cyclicity reflects the functional integrity of the hypothalamic–pituitary–ovarian axis and cyclic changes in ovarian steroid secretion, estrous-cycle monitoring was included as a functional assessment of female reproductive endocrine regulation and to determine whether paroxetine exposure had altered this regulation before the initiation of irisin treatment [34,38].

### 2.5. Irisin Administration Protocol

After 4 weeks of paroxetine treatment, following the identification of paroxetine-associated estrous-cycle irregularity, irisin treatment was initiated in the irisin and paroxetine + irisin groups. Irisin (SRP8039; Sigma-Aldrich, St. Louis, MO, USA) was continuously administered at a dose of 100 ng/kg/day in deionized water for 28 days using subcutaneously implanted mini-osmotic pumps (Alzet model 2004; Durect Corporation, Cupertino, CA, USA). The irisin dose was selected based on our previous study [28], in which 100 ng/kg/day produced measurable reproductive and endocrine effects in female rats, supporting the biological activity of this dose. Animals in the sham control group underwent the same implantation procedure using identical pumps filled with deionized water alone. The paroxetine-only group did not undergo anesthesia, sham surgery, or implantation of a vehicle-filled mini-osmotic pump.

The mini-osmotic pumps had a flow rate of 0.25 μL/h, a reservoir capacity of 200 μL, and an operating duration of 28 days. Before implantation, rats undergoing the implantation procedure were anesthetized with an intraperitoneal injection of 60 mg/kg ketamine and 5 mg/kg xylazine cocktail. Under aseptic conditions, each pump, containing either 200 μL of 6 ng/μL irisin or an equivalent volume of deionized water, was implanted subcutaneously in the interscapular region. This implantation site was selected to minimize interference with normal activity. Pump function was verified by comparing the reservoir volume before implantation with the residual volume following pump removal on day 28, confirming continuous delivery of irisin or vehicle throughout the treatment period. The group-specific treatment and procedural control schedule is summarized in Table 1.

### 2.6. Sexual Incentive Motivation (SIM) Test

#### 2.6.1. SIM Apparatus

The sexual incentive motivation (SIM) apparatus consisted of a transparent rectangular Plexiglas box measuring 91.44 × 31.75 × 40 cm as described in our previous study [29]. The apparatus comprised a central compartment and two lateral stimulus compartments, each 16.51 cm wide. The lateral compartments were separated from the central compartment by transparent partitions containing 1 cm-diameter perforations, allowing visual, olfactory, and auditory interaction while preventing direct physical contact between the experimental female and the stimulus animals. On the day of the SIM test, the sexual and social stimulus animals were placed individually in their respective lateral compartments, and one experimental female from each group was introduced into the central compartment. A dashed black line at the midpoint of the central compartment divided it into two equal zones and was used to assess the activity of the experimental female.

#### 2.6.2. Habituation Procedure

One week before the SIM or active investigation test, the experimental female rats were habituated to the testing apparatus as described in our previous study [29]. Habituation consisted of two sessions in which each rat was placed individually in the central compartment and allowed to explore freely for 30 min in the absence of stimulus animals. The sexual stimulus animal was a sexually experienced, gonadally intact adult male rat, whereas the social stimulus was an ovariectomized adult female rat.

#### 2.6.3. SIM Test Protocol

Following approximately 3 weeks of sustained irisin infusion, the SIM and active investigation tests were conducted as described in our previous study [29]. Before behavioral testing, vaginal smears were collected and examined to determine the estrous cycle stage. Only females identified as being in behavioral estrus, defined as the estrus phase of the estrous cycle in which the female is sexually receptive to the male, were included in the SIM test [39,40]. At the beginning of each session, the experimental female was introduced into the central compartment and allowed to explore and adapt to the apparatus for 5 min. The female was then returned to its home cage while the sexual and social stimulus animals were placed in their designated lateral compartments. Subsequently, the experimental female was reintroduced into the central compartment, and the 10 min test recording was initiated. During the 10 min test, the time spent near each stimulus animal was recorded. The experimental female was considered to be near the sexual or social stimulus when all four paws were located within the 12.7 cm zone adjacent to the partition separating the central compartment from the respective stimuli. Accordingly, TWM, TWF, TST (TWM + TWF), and the male preference ratio (TWM/TST) were determined. In addition, the activity was evaluated as the total number of crossings through the center of the test apparatus over 10 min and was scored manually using a hand counter.

#### 2.6.4. Active Investigation Assessment

Active investigation behaviors were evaluated simultaneously with the SIM test in the same apparatus, following the approach described in a previous study [41]. Active investigation is characterized by experimental female behaviors directed toward the barriers separating it from the stimulus animals, including sniffing, licking, chewing, and climbing, in an apparent attempt to reach the sexual or social stimulus. Over the 10 min observation period, TIM, TIF, TIT (TIM + TIF), and the male investigation preference ratio (TIM/TIT) were recorded. Video recordings of both the SIM and active investigation tests were scored manually with a stopwatch by the same experienced observer, who was blinded to the experimental groups and applied predefined behavioral criteria consistently across all recordings.

### 2.7. Experimental Termination and Sample Collection

Following completion of the SIM and active investigation tests, which were conducted after approximately 3 weeks of sustained irisin infusion, the estrous stage was determined by vaginal cytology to minimize potential estrous-cycle-related variability in circulating reproductive hormone levels [42]. During the fourth week of the 28-day irisin infusion period, each animal was euthanized by rapid decapitation without prior anesthesia once diestrus had been confirmed, and blood and tissue samples were collected. Blood samples were subsequently centrifuged at 4000 rpm for 5 min at 4 °C, and the separated serum was stored at −80 °C until enzyme-linked immunosorbent assay (ELISA) analysis.

### 2.8. ELISA Analyses

Serum levels of testosterone (Enzo Life Sciences AG, Lausen, Switzerland; Cat. No. ADI-900-065), LH (Enzo Life Sciences AG, Lausen, Switzerland; ENZ-KIT107), FSH (Elabscience Biotechnology Inc, Houston, TX, USA; Cat. No. E-EL-R0391), and AMH (Elabscience Biotechnology Inc, Houston, TX, USA; Cat. No. E-EL-R0640) were measured according to the manufacturer’s instructions using a Multiskan FC microplate reader (Thermo Fisher Scientific, Waltham, MA, USA). All samples were analyzed in duplicate. The assay sensitivity and detection range were 5.67 pg/mL and 7.81–2.000 pg/mL for testosterone, 5.2 mIU/mL and 1.2–280 mIU/mL for LH, 1.88 ng/mL and 3.13–200 ng/mL for FSH, and 0.094 ng/mL and 0.156–10 ng/mL for AMH, respectively.

### 2.9. Histological Analyses

For histological and immunohistochemical evaluations, vaginal tissues were excised and then fixed in 10% formalin. Following routine histological processing, the tissues were dehydrated and embedded in paraffin. From each paraffin block, five serial cross-sections (5 μm thick) were obtained at 30 μm intervals. The sections were mounted on glass slides, and stained with hematoxylin and eosin (H&E), methyl green–pyronin (Histomed, Ankara, Turkey, BS-366, Lot: 102023.018), and periodic acid–Schiff (PAS). H&E-stained sections were used for fibroblast counting, methyl green–pyronin-stained sections for plasma cell counting, and PAS-stained sections for the assessment of vaginal epithelial glycogen content.

All sections were assessed under 10× magnification and photographed using a microscope (Nikon Eclipse 50i, Tokyo, Japan). For each animal, histological evaluations were performed across all prepared sections, and mean values were calculated for each parameter within each group. For all experimental groups, fibroblasts and plasma cells were quantified in 10 randomly selected areas from H&E- and methyl green–pyronin-stained vaginal sections, respectively. Cell counting was performed at ×40 magnification using a 100-square ocular micrometer (eyepiece graticule), and the results were expressed as the number of cells per square millimeter (cells/mm^2^) [43]. In addition, PAS reactivity in the vaginal epithelium was semiquantitatively graded according to staining intensity as none (−), weak (+), moderate (++), or severe (+++), as in our previous study [44].

### 2.10. Immunohistochemical Analysis of TLR4 and NF-κB in Vaginal Tissue

Vaginal TLR4 and NF-κB expression and localization were evaluated immunohistochemically as previously described [45]. Briefly, five vaginal sections were obtained from each paraffin block at 30 μm intervals, assessed for TLR4 and NF-κB immunoreactivity, and the mean value was calculated for each animal. Following deparaffinization and antigen retrieval, the sections were incubated overnight at 4 °C with either a mouse monoclonal TLR4 antibody (1:300; Santa Cruz Biotechnology, Dallas, TX, USA; catalog no. sc-293072) or a mouse monoclonal NF-κB antibody (1:500; Santa Cruz Biotechnology; catalog no. sc-8008). Immunoreactivity was determined using a goat anti-polyvalent horseradish peroxidase-conjugated antibody (Patolab, Istanbul, Turkey, PL-125-HL) and 3,3′-diaminobenzidine chromogen (Patolab, Istanbul, Turkey, PL-125-HD), in accordance with the manufacturer’s instructions. The sections were subsequently counterstained with hematoxylin, mounted with Entellan, and examined under a light microscope.

TLR4 and NF-κB immunoreactivity were evaluated separately using a histoscore method at 20× magnification. For each marker, both immunostaining intensity and the percentage of the immunopositive tissue area were assessed. Immunostaining intensity was graded as negative (0), trace (0.5), light (1), moderate (2), or intense (3). The percentage of the immunopositive area was scored as 0 for no staining, 0.1 for <25%, 0.4 for 26–50%, 0.6 for 51–75%, and 0.9 for 76–100% staining, indicating near-homogeneity. A composite histoscore, incorporating both staining intensity and the uniformity of the immunopositive area, was calculated by multiplying the mean intensity value by the mean percentage of stained area (histoscore = intensity × area) [44]. For each animal, five serial cross-sections of 5 μm thickness were obtained from each block at 30 μm intervals. Histoscores obtained from the assessed areas were averaged to calculate a single mean histoscore for each animal. All immunohistochemical assessments were conducted by an experienced observer blinded to the experimental groups.

### 2.11. Statistical Analysis

The normality of the residuals was assessed using Q–Q plots and the Shapiro–Wilk test, and homogeneity of variances was evaluated using Levene’s test. For variables for which these assessments supported the use of parametric analysis, group differences were analyzed using one-way analysis of variance (ANOVA), followed by Tukey’s HSD post hoc test for pairwise comparisons. Differences in the frequency of irregular estrous cyclicity between paroxetine-exposed and non-paroxetine-exposed animals were analyzed using Pearson’s chi-square test. Eta-squared (η^2^) was calculated to quantify the magnitude of the group effects. Values of η^2^ ≥ 0.01, ≥ 0.06, and ≥ 0.14 were interpreted as small, medium, and large effects, respectively. For post hoc pairwise comparisons, 95% confidence intervals (CIs) and adjusted *p* values were reported. Results are expressed as the mean ± standard error of the mean (SEM). All statistical analyses were performed using SPSS software, version 27.0 (IBM Corp., Armonk, NY, USA), and graphical representations were generated using GraphPad Prism version 8.0.1 for Windows (GraphPad Software, San Diego, CA, USA). Statistical significance was defined as *p* < 0.05.

## 3. Results

### 3.1. Estrous-Cycle Regularity Before Irisin Treatment

Before the initiation of irisin treatment, irregular estrous cyclicity was observed in 14 of 20 (70%) paroxetine-exposed rats, whereas none of the 20 non-paroxetine-exposed rats exhibited irregular cyclicity. Pearson’s chi-square analysis showed that the frequency of irregular estrous cyclicity was significantly higher in paroxetine-exposed animals than in non-paroxetine-exposed animals [χ^2^(1) = 21.538, *p* = 0.000003].

### 3.2. SIM and Active Investigation Test

**Figure 1:**
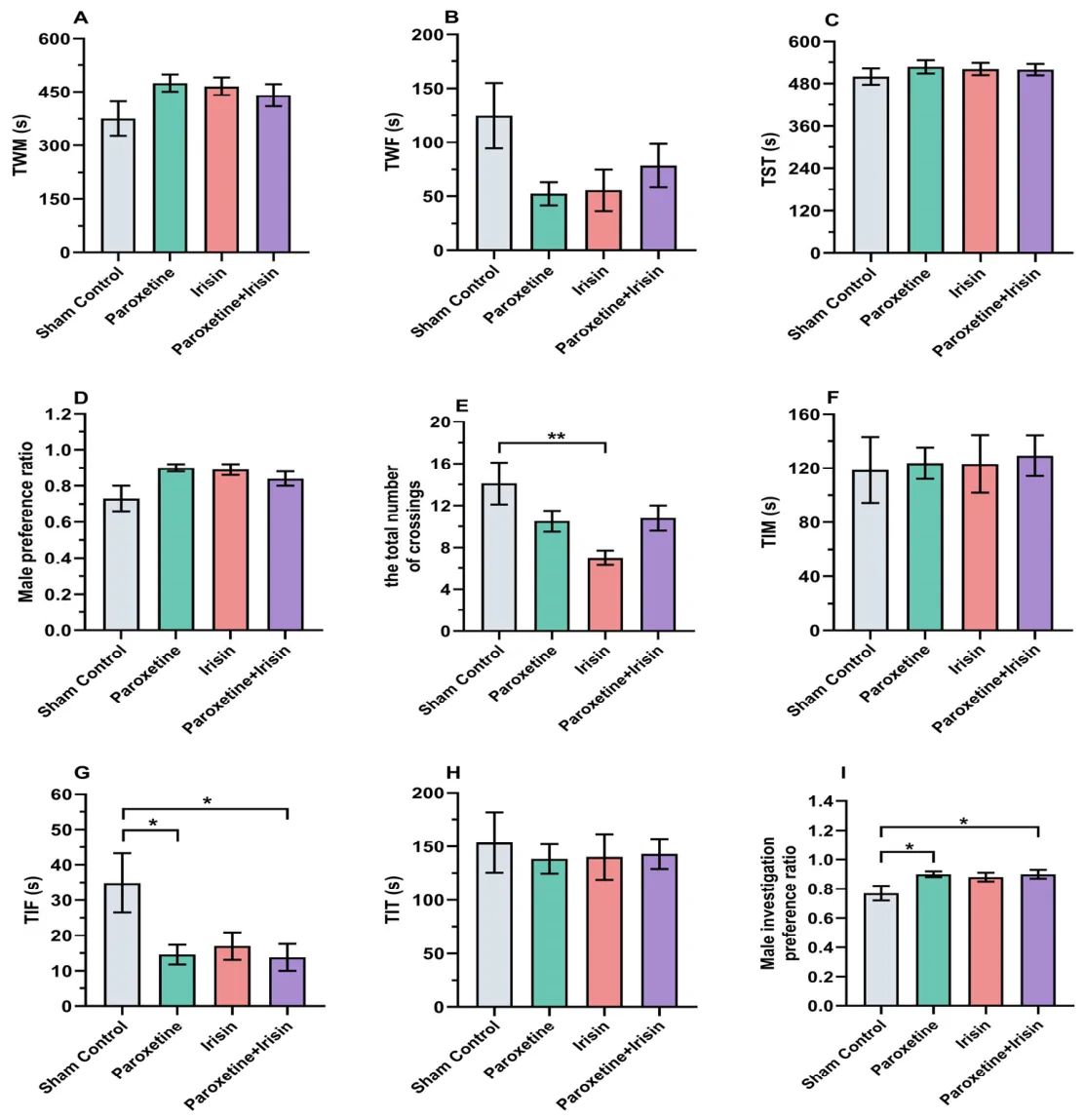
Effects of paroxetine and/or irisin treatment on sexual incentive motivation, activity, and active investigation behavior in adult female rats. (**A**) TWM, (**B**) TWF, (**C**) TST, (**D**) male preference ratio, (**E**) the total number of crossings, (**F**) TIM, (**G**) TIF, (**H**) TIT, and (**I**) male investigation preference ratio (Data are presented as mean ± SEM and were analyzed using one-way ANOVA. * *p* < 0.05 and ** *p* < 0.01 vs. the sham control group; *n* = 10 per group).

Figure 1A–I illustrates the results of the SIM and active investigation tests. One-way ANOVA revealed no statistically significant group differences in TWM, TWF, TST, male preference ratio, TIM, or TIT (*p* > 0.05). However, the activity was significantly lower in the irisin group than in the sham control group (Figure 1E, *p* < 0.01). In the active investigation test, both the paroxetine and paroxetine + irisin groups exhibited significantly lower TIF and higher male investigation preference ratios than the sham control group (Figure 1G and I, respectively; *p* < 0.05).

### 3.3. Reproductive Hormonal Profile

**Figure 2:**
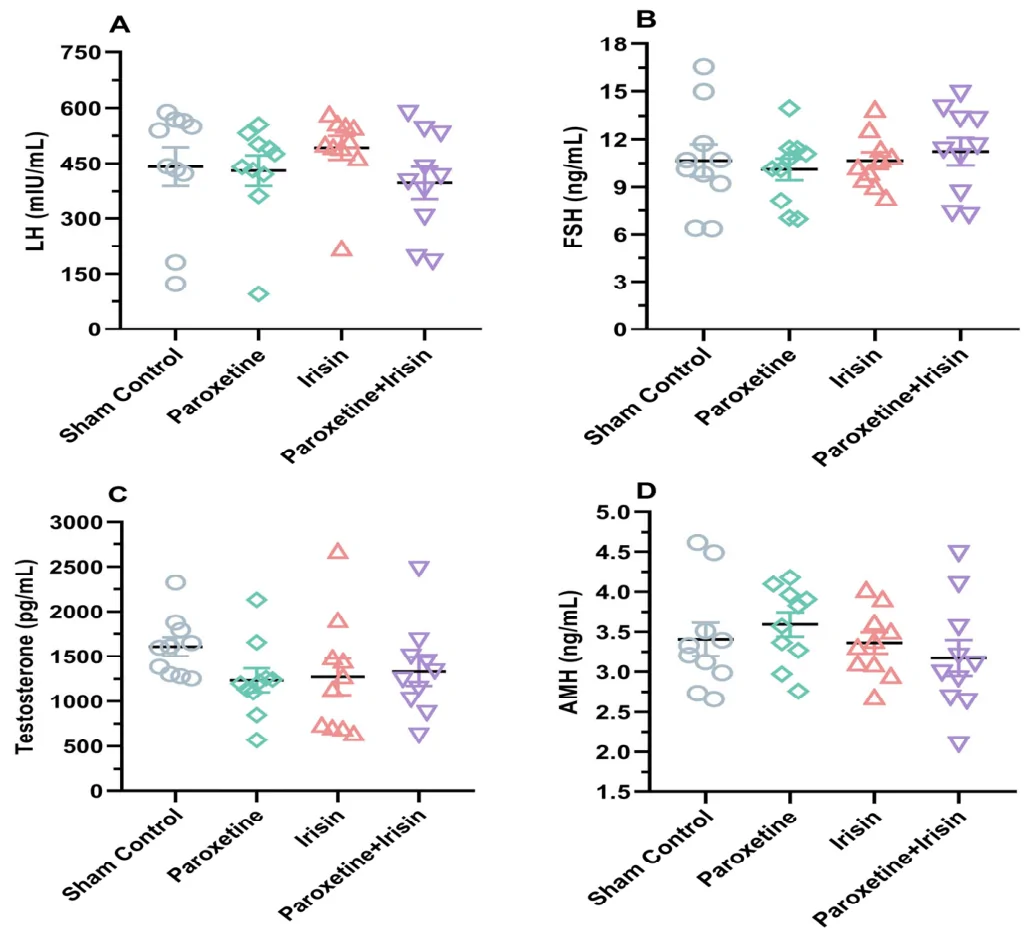
Effects of paroxetine and/or irisin treatment on serum reproductive hormone concentrations in adult female rats: (**A**) LH, (**B**) FSH, (**C**) testosterone, and (**D**) AMH (Data are presented as mean ± SEM and were analyzed using one-way ANOVA followed by Tukey’s HSD post hoc test. *n* = 10 per group).

Figure 2A–D presents the serum concentrations of LH, FSH, testosterone, and AMH across the experimental groups. One-way ANOVA revealed no statistically significant between-group differences in serum LH [*F*(3,36) = 0.840, *p* = 0.481, η^2^ = 0.065, Figure 2A], FSH [*F*(3,36) = 0.333, *p* = 0.802, η^2^ = 0.027, Figure 2B], testosterone [*F*(3,36) = 1.175, *p* = 0.333, η^2^ = 0.089, Figure 2C], or AMH [*F*(3,36) = 0.873, *p* = 0.464, η^2^ = 0.068, Figure 2D]. Consistent with the overall ANOVA results, Tukey’s post hoc analysis revealed no significant pairwise differences in LH: sham control vs. paroxetine (95% CI: −153.13 to 174.89, *p* = 0.998), sham control vs. irisin (95% CI: −215.22 to 112.80, *p* = 0.835), sham control vs. paroxetine + irisin (95% CI: −120.01 to 208.01, *p* = 0.887), paroxetine vs. paroxetine + irisin (95% CI: −130.89 to 197.13, *p* = 0.948), and irisin vs. paroxetine + irisin (95% CI: −68.80 to 259.22, *p* = 0.412). Similarly, no significant pairwise differences were observed for FSH: sham control vs. paroxetine (95% CI: −2.49 to 3.60, *p* = 0.961), sham control vs. irisin (95% CI: −3.04 to 3.04, *p* = 1.000), sham control vs. paroxetine + irisin (95% CI: −3.62 to 2.47, *p* = 0.956), paroxetine vs. paroxetine + irisin (95% CI: −4.17 to 1.92, *p* = 0.751), and irisin vs. paroxetine + irisin (95% CI: −3.62 to 2.47, *p* = 0.956). No significant pairwise differences were detected for testosterone: sham control vs. paroxetine (95% CI: −219.74 to 965.40, *p* = 0.341), sham control vs. irisin (95% CI: −258.22 to 926.92, *p* = 0.437), sham control vs. paroxetine + irisin (95% CI: −316.02 to 869.12, *p* = 0.596), paroxetine vs. paroxetine + irisin (95% CI: −688.85 to 496.29, *p* = 0.972), and irisin vs. paroxetine + irisin (95% CI: −650.37 to 534.77, *p* = 0.994). Tukey’s post hoc comparisons were also nonsignificant for AMH: sham control vs. paroxetine (95% CI: −0.89 to 0.51, *p* = 0.889), sham control vs. irisin (95% CI: −0.66 to 0.74, *p* = 0.998), sham control vs. paroxetine + irisin (95% CI: −0.47 to 0.93, *p* = 0.809), paroxetine vs. paroxetine + irisin (95% CI: −0.28 to 1.12, *p* = 0.386), and irisin vs. paroxetine + irisin (95% CI: −0.51 to 0.89, *p* = 0.886).

### 3.4. Histological and Immunohistochemical Findings in Vaginal Tissue

**Figure 3:**
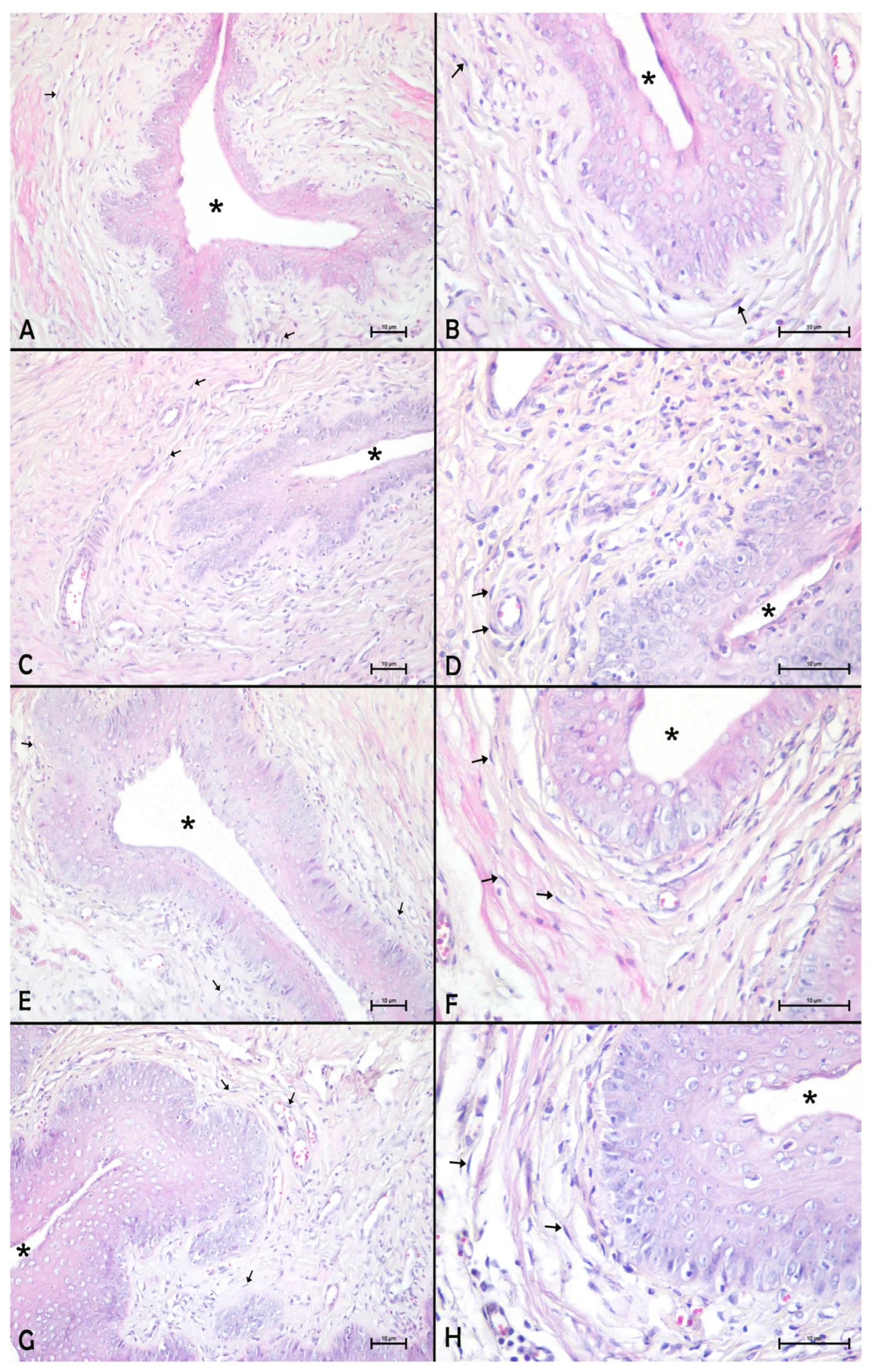
Representative vaginal sections showing fibroblasts following paroxetine and/or irisin treatment, stained with hematoxylin and eosin (H&E). (**A**,**B**) Sham control, (**C**,**D**) paroxetine, (**E**,**F**) irisin, and (**G**,**H**) paroxetine + irisin groups. Panels (**A**,**C**,**E**,**G**) are shown at 20× magnification, whereas panels (**B**,**D**,**F**,**H**) are shown at 40× magnification. The asterisk indicates the vaginal lumen, and the arrows indicate fibroblasts. Scale bar = 10 μm.

**Figure 4:**
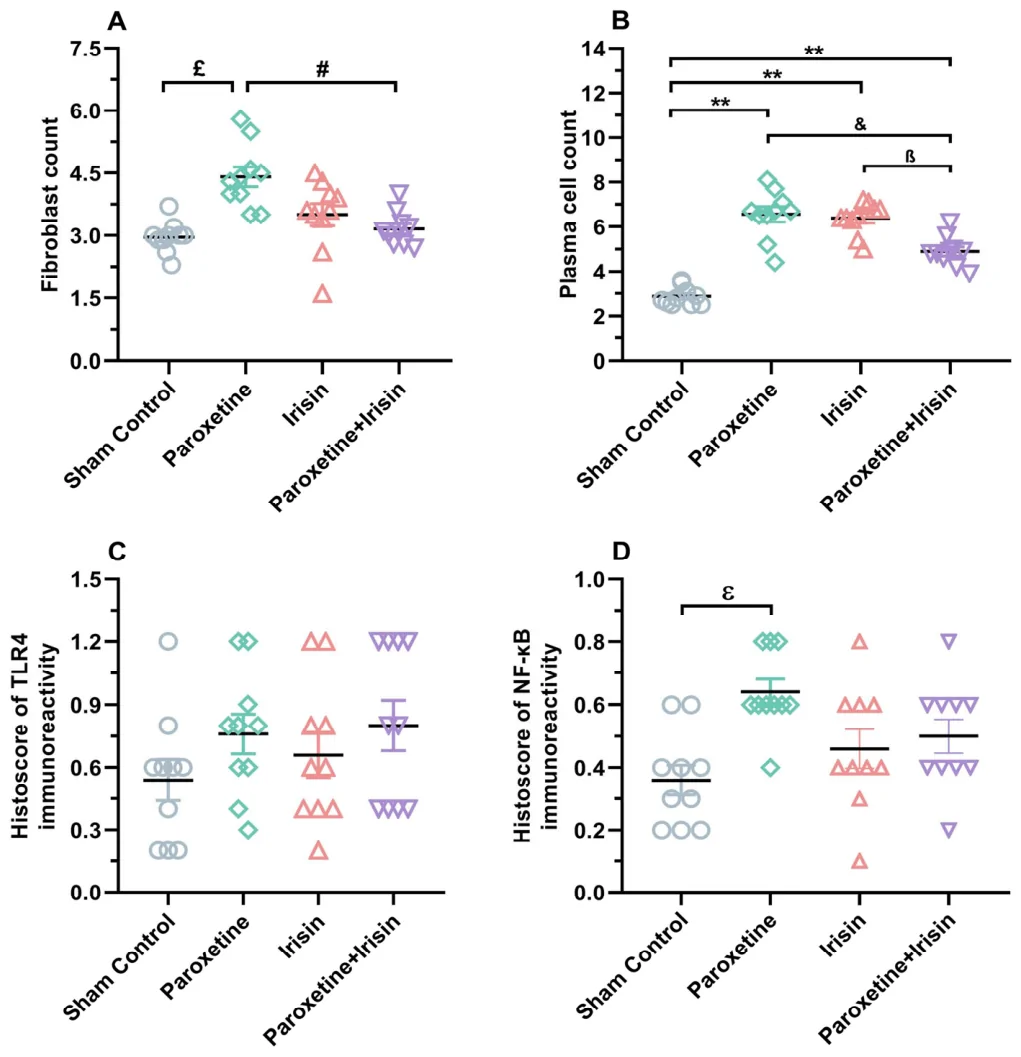
Effects of paroxetine and/or irisin treatment on vaginal (**A**) fibroblast cell count, (**B**) plasma cell count, (**C**) TLR4 immunoreactivity histoscore, and (**D**) NF-κB immunoreactivity histoscore in adult female rats. Bar graph data are presented as mean ± SEM and were analyzed using one-way ANOVA followed by Tukey’s HSD post hoc test. £: *p* = 0.0001; *#*: *p* = 0.0005; **: *p* < 0.0001; &: *p* = 0.0001; ß: *p* = 0.0004; ε: *p* = 0.003; *n* = 10 per group.

Figure 3A–H presents representative vaginal sections showing fibroblasts across the experimental groups. The one-way ANOVA results showing the effects of paroxetine and/or irisin treatment on vaginal fibroblast cell counts are presented in Figure 4A. A significant overall group effect was observed for fibroblast counts [*F*(3,36) = 10.381, *p* = 0.000046, η^2^ = 0.464, Figure 4A]. Tukey’s post hoc analysis showed that fibroblast count was significantly higher in the paroxetine group than in the sham control group (95% CI: −2.2085 to −0.6915, *p* = 0.0001), whereas it was significantly lower in the paroxetine + irisin group than in the paroxetine group (95% CI: 0.4915 to 2.0085, *p* = 0.0005). No significant differences were observed between the sham control and irisin groups (95% CI: −1.2985 to 0.2185, *p* = 0.239), sham control and paroxetine + irisin groups (95% CI: −0.9585 to 0.5585, *p* = 0.892), or irisin and paroxetine + irisin groups (95% CI: −0.4185 to 1.0985, *p* = 0.626).

**Figure 5:**
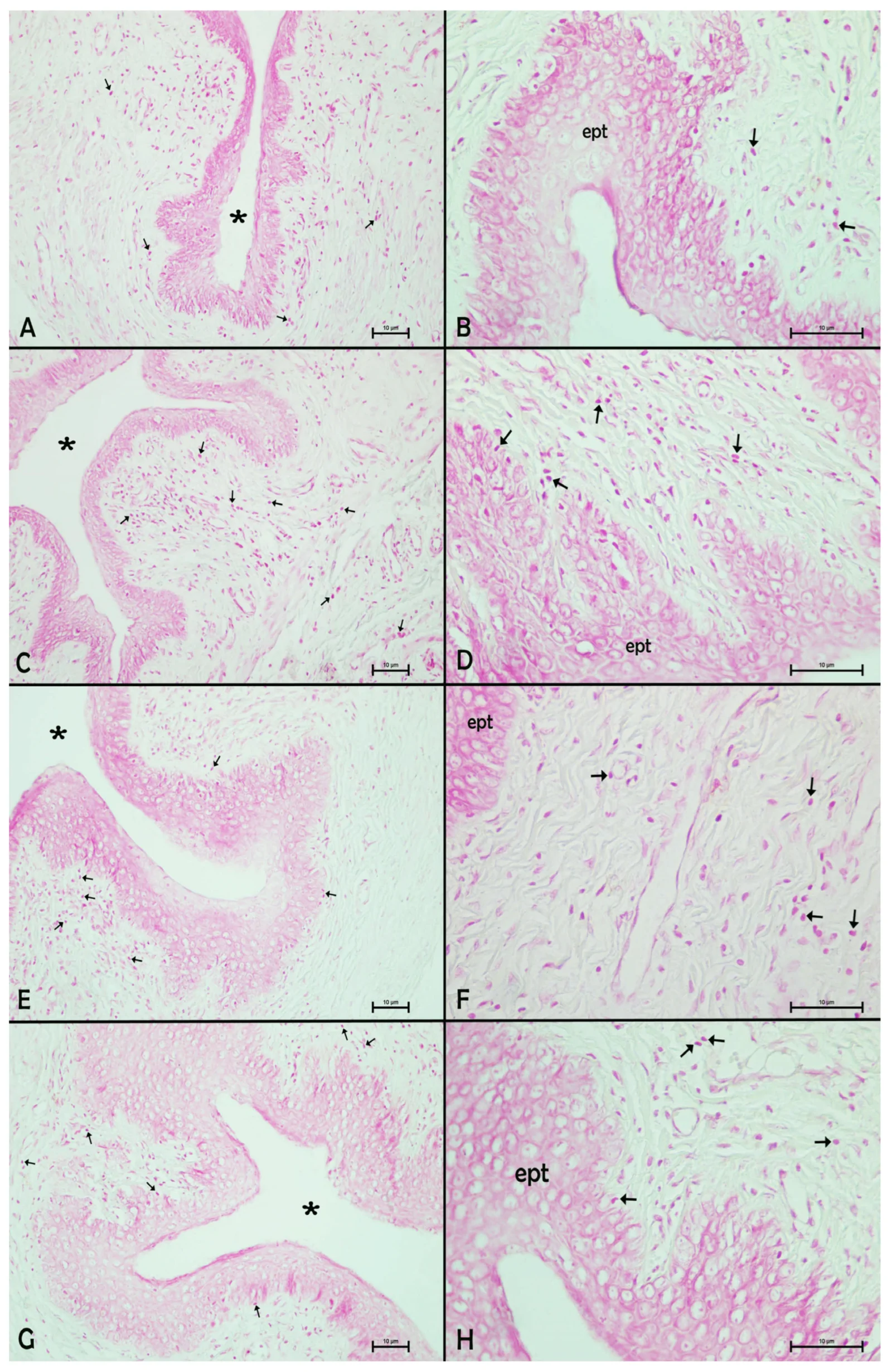
Representative vaginal sections showing plasma cells following paroxetine and/or irisin treatment, stained with methyl green–pyronin. (**A**,**B**) Sham control, (**C**,**D**) paroxetine, (**E**,**F**) irisin, and (**G**,**H**) paroxetine + irisin groups. Panels (**A**,**C**,**E**,**G**) are shown at 20× magnification, whereas panels (**B**,**D**,**F**,**H**) are shown at 40× magnification. The asterisk indicates the vaginal lumen, “ept” indicates the epithelium, and the arrows indicate plasma cells. Scale bar = 10 μm.

Figure 5A–H illustrates plasma cells in vaginal tissue across the experimental groups. One-way ANOVA revealed a significant overall group effect for vaginal plasma cell counts [*F*(3,36) = 52.048, *p* < 0.0001, η^2^ = 0.813, Figure 4B]. Tukey’s post hoc analysis showed significantly higher plasma cell counts in the paroxetine (95% CI: −4.5936 to −2.7864, *p* < 0.0001), irisin (95% CI: −4.4236 to −2.6164, *p* < 0.0001), and paroxetine + irisin groups (95% CI: −2.9236 to −1.1164, *p* < 0.0001) than in the sham control group. Plasma cell counts were significantly lower in the paroxetine + irisin group than in both the paroxetine (95% CI: 0.7664 to 2.5736, *p* = 0.0001) and irisin groups (95% CI: 0.5964 to 2.4036, *p* = 0.0004).

**Figure 6:**
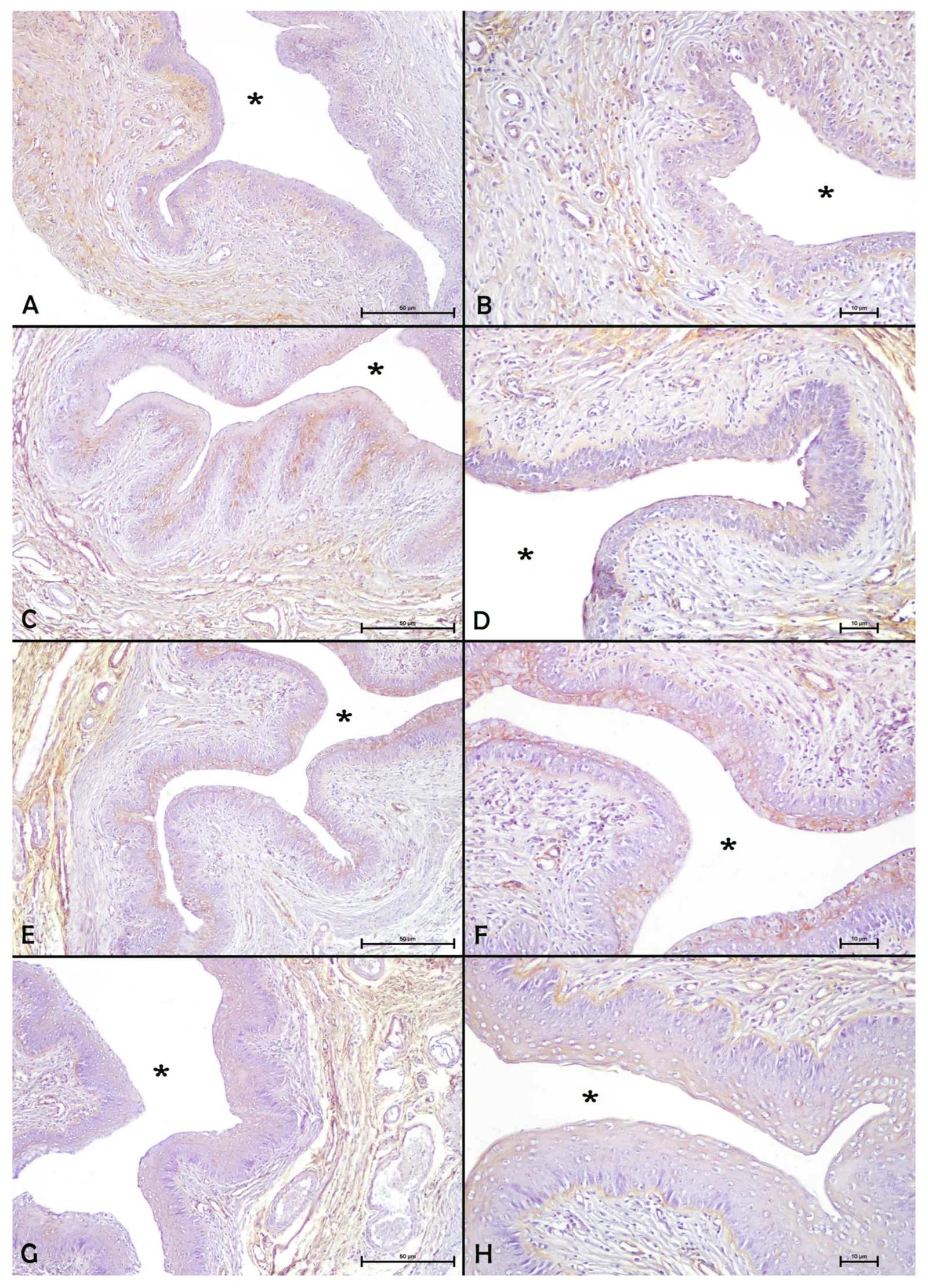
Representative vaginal sections illustrating TLR4 immunoreactivity following paroxetine and/or irisin treatment. (**A**,**B**) Sham control, (**C**,**D**) paroxetine, (**E**,**F**) irisin, and (**G**,**H**) paroxetine + irisin groups. Panels (**A**,**C**,**E**,**G**) are shown at 10× magnification, whereas panels (**B**,**D**,**F**,**H**) are shown at 20× magnification. Brown staining indicates TLR4 immunoreactivity, and the asterisk indicates the vaginal lumen. Scale bars = 10 μm and 50 μm.

TLR4 immunoreactivity in vaginal sections is shown in Figure 6A–H. No significant overall group effect was observed for vaginal TLR4 immunoreactivity histoscores [*F*(3,36) = 1.208, *p* = 0.321, η^2^ = 0.091, Figure 4C]. Tukey’s post hoc comparisons were nonsignificant for sham control vs. paroxetine (95% CI: −0.6221 to 0.1821, *p* = 0.464), sham control vs. irisin (95% CI: −0.5221 to 0.2821, *p* = 0.852), sham control vs. paroxetine + irisin (95% CI: −0.6621 to 0.1421, *p* = 0.318), paroxetine vs. paroxetine + irisin (95% CI: −0.4421 to 0.3621, *p* = 0.993), and irisin vs. paroxetine + irisin (95% CI: −0.5421 to 0.2621, *p* = 0.785).

**Figure 7:**
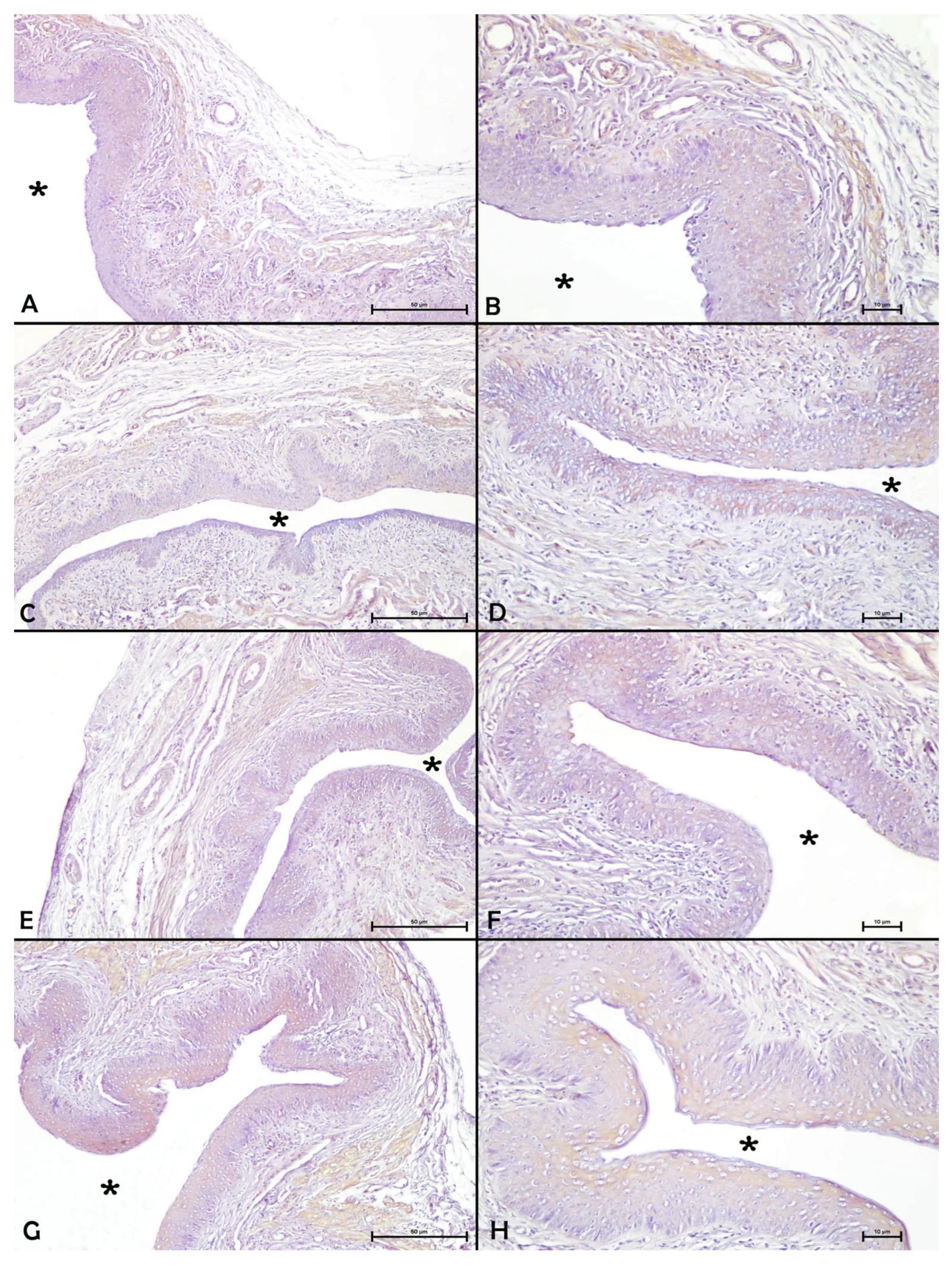
Representative vaginal sections illustrating NF-κB immunoreactivity following paroxetine and/or irisin treatment. (**A**,**B**) Sham control, (**C**,**D**) paroxetine, (**E**,**F**) irisin, and (**G**,**H**) paroxetine + irisin groups. Panels (**A**,**C**,**E**,**G**) are shown at 10× magnification, whereas panels (**B**,**D**,**F**,**H**) are shown at 20× magnification. Brown staining indicates NF-κB immunoreactivity, and the asterisk indicates the vaginal lumen. Scale bars = 10 μm and 50 μm.

**Figure 8:**
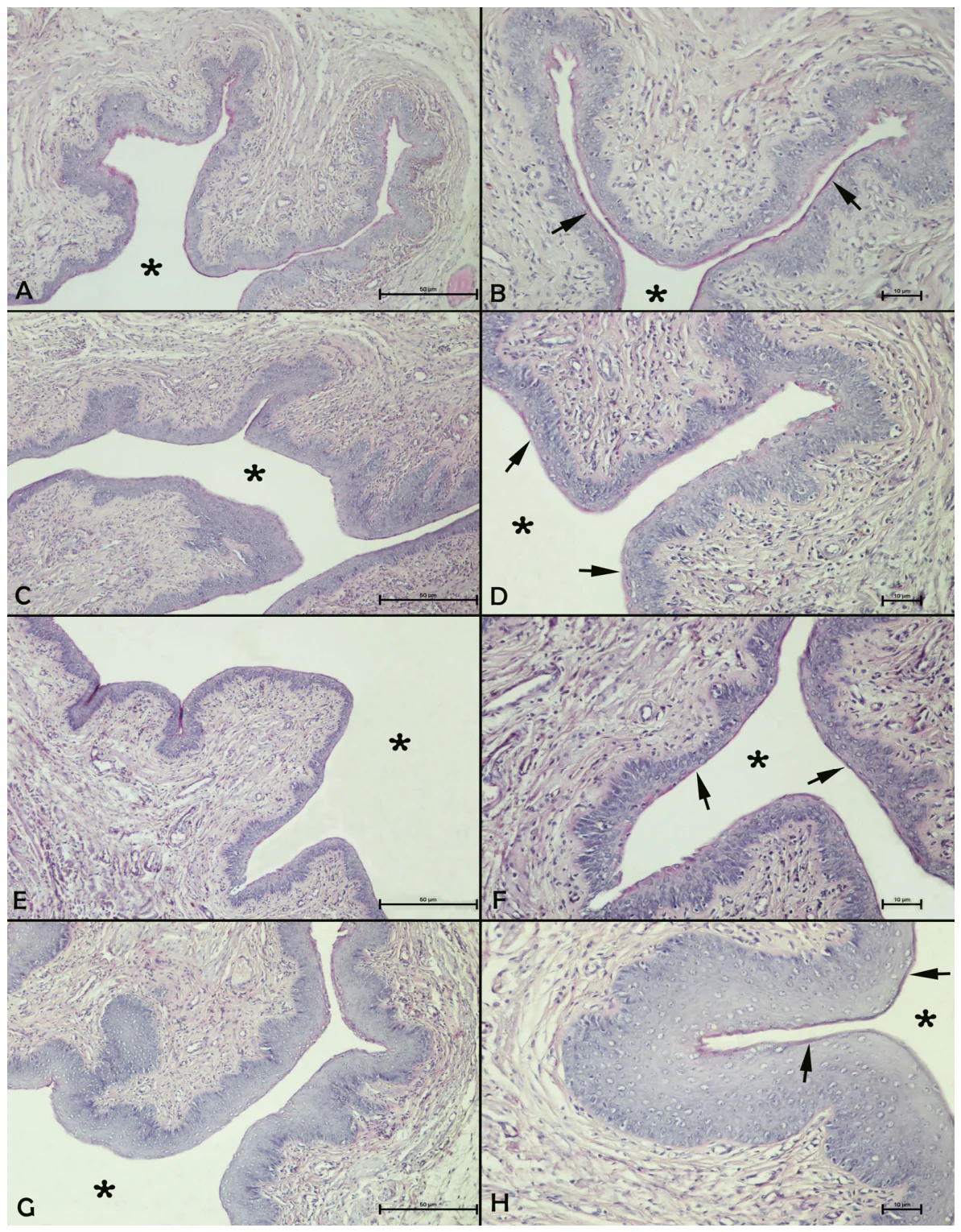
Representative periodic acid–Schiff (PAS)-stained vaginal sections following paroxetine and/or irisin treatment. (**A**,**B**) Sham control, (**C**,**D**) paroxetine, (**E**,**F**) irisin, and (**G**,**H**) paroxetine + irisin groups. Panels (**A**,**C**,**E**,**G**) are shown at 10× magnification, whereas panels (**B**,**D**,**F**,**H**) are shown at 20× magnification. The asterisk indicates the vaginal lumen, and the arrows indicate PAS-positive staining in the apical regions of epithelial cells facing the lumen. Scale bars = 10 μm and 50 μm.

NF-κB immunoreactivity is presented in Figure 7A–H. A significant overall group effect was observed for vaginal NF-κB immunoreactivity histoscores [*F*(3,36) = 5.092, *p* = 0.004849, η^2^ = 0.298, Figure 4D]. Tukey’s post hoc analysis showed significantly higher NF-κB immunoreactivity in the paroxetine group than in the sham control group (95% CI: −0.4759 to −0.0841, *p* = 0.003). No significant differences were observed between the sham control and irisin groups (95% CI: −0.2959 to 0.0959, *p* = 0.523), sham control and paroxetine + irisin groups (95% CI: −0.3359 to 0.0559, *p* = 0.236), paroxetine and paroxetine + irisin groups (95% CI: −0.0559 to 0.3359, *p* = 0.236), or irisin and paroxetine + irisin groups (95% CI: −0.2359 to 0.1559, *p* = 0.946). Regarding PAS reactivity in the vaginal epithelium, severe (+++) staining was observed in the sham control group, weak (+) staining in the paroxetine group, and moderate (++) staining in both the irisin and paroxetine + irisin groups (Figure 8A–H, Table 2).

## 4. Discussion

The present study investigated the effects of chronic paroxetine exposure and/or irisin administration on female reproductive function. Paroxetine altered active investigation behavior, increased vaginal fibroblast counts and NF-κB immunoreactivity, and reduced epithelial PAS reactivity, while serum reproductive hormones and TLR4 immunoreactivity remained unchanged. Irisin alone reduced activity and increased plasma cell counts. In the combined-treatment group, the behavioral alterations persisted; however, fibroblast and plasma cell counts were reduced, and epithelial PAS reactivity was partially preserved.

### 4.1. Behavioral and Endocrine Effects on Female Reproductive Function

Sexual incentive motivation is evaluated using the SIM test in rats. It is defined as the time spent in an area near an inaccessible sexual stimulus relative to the time spent in an area near an inaccessible social stimulus [41]. In this paradigm, sexually receptive females, either naturally in proestrus or hormonally primed with estradiol and progesterone, typically display a greater preference for a sexually active male than females in diestrus or ovariectomized animals [46].

In the present study, paroxetine did not alter TWM, TWF, TST, or the male preference ratio. By contrast, both the paroxetine and paroxetine + irisin groups exhibited lower TIF and consequently higher male investigation preference ratios, without a significant increase in TIM. Thus, irisin did not change the paroxetine-associated alterations in active investigation behavior. In addition, activity was significantly lower in the irisin group than in the sham control group. Few studies have directly investigated the effects of paroxetine or irisin on sexual incentive motivation in female rats. Kaspersen and Ågmo (2012) reported that chronic paroxetine reduced sexual incentive motivation in ovariectomized, hormonally primed adult female rats [7]. However, their experimental model differed from that used in the present study. Whereas they employed a gonadally intact male and a castrated male as the sexual and social incentives, respectively, the present study used a gonadally intact male and an ovariectomized female rat. Moreover, hormonal priming in the previous study, together with differences in paroxetine dose, route of administration, and treatment duration, may have contributed to the different findings. Consistent with the preserved SIM outcomes observed here, our previous study showed that approximately four weeks of oral paroxetine administration did not alter TWM, TWF, TST, or the male preference ratio in adult female rats [29]. Active investigation parameters, including TIM, TIF, TIT, and the male investigation preference ratio, were also unaffected in that study. Despite differences in dose and treatment duration, paroxetine did not alter TWM, TWF, TST, or the male preference ratio in either study. However, the lower TIF and higher male investigation preference ratio observed only in the present study suggest that prolonged paroxetine exposure may selectively alter investigation directed toward the female social stimulus without affecting sexual incentive motivation. Using a different SSRI and a similar partner-preference paradigm with a gonadally intact male and an ovariectomized female as incentives, Adams et al. (2012) found that acute fluoxetine treatment increased TWF and reduced TIM and the male investigation preference ratio, while the male preference ratio remained unchanged in ovariectomized, hormonally primed female rats [47]. These findings differ from the present results, in which prolonged paroxetine exposure was associated with lower TIF and a higher male investigation preference ratio, suggesting that the pattern of investigation behavior may vary according to the SSRI used, treatment duration, and experimental conditions [47]. More recent evidence further demonstrates that serotonergic signaling contributes to the regulation of social behavior in rats. Reduced serotonin transporter function and the resulting increase in extracellular serotonin have been associated with reduced social contact in rats [48]. More recently, higher amygdala serotonin levels were negatively associated with social investigation and interaction [49]. In this context, these findings suggest that the reduced investigation of the female social stimulus in the present study may be, at least in part, attributable to possible enhanced serotonergic neurotransmission during prolonged paroxetine exposure.

When the effects of irisin on activity were assessed, irisin treatment alone was observed to significantly reduce locomotor activity. In our previous study, chronic daily intraperitoneal administration of irisin at 100 ng/kg from the postweaning period for approximately 10 weeks significantly increased the number of crossings in female rats [29]. Similarly, Zhang et al. (2015) reported that a single third-ventricular intracerebroventricular (ICV) administration of irisin at 1.5 or 2.5 μg/kg produced a rapid and transient increase in locomotor activity in male rats, whereas peripheral administration had no detectable effect [50]. In contrast, a single ICV administration of irisin at 0.1, 0.5, and 1 ng/mouse did not alter locomotor activity in male mice [51]. Likewise, once-daily subcutaneous administration of irisin at 100 μg/kg for five consecutive days did not significantly alter locomotor activity in male or female mice [52]. More recently, Inyushkin et al. (2025) demonstrated that three intranasal administrations of irisin at 0.5 μg significantly reduced voluntary locomotor activity in adult male rats, with the response depending on the timing of administration [53]. Taken together, these findings indicate that the locomotor response to exogenous irisin is not uniform across experimental models and may be influenced by differences in dose, route and regimen of administration, duration of exposure, and the age and sex of the animals. The time-dependent effect reported by Inyushkin et al. (2025) further suggests that the timing of irisin administration may also contribute to variability in locomotor responses [53]. In this context, the differing activity outcomes may partly reflect differences in the age at treatment initiation, route and regimen of administration, and treatment duration. In the present study, irisin was administered at 100 ng/kg/day by continuous subcutaneous infusion for four weeks in adult rats. In contrast, in our previous study, the same dose was administered by daily intraperitoneal injection from the postweaning period for approximately 10 weeks. Given the reduction in locomotor activity observed with irisin alone, this finding should also be considered when interpreting the behavioral results, as general activity may influence approach-based measures. However, irisin did not alter TWM, TWF, TST, or the male preference ratio, suggesting that this reduction may not have substantially affected sexual incentive motivation. Moreover, active investigation changes were observed in the paroxetine-treated groups, whereas reduced locomotor activity occurred only with irisin alone, suggesting that decreased general activity may not fully account for these behavioral findings.

Regarding reproductive endocrine outcomes, no significant between-group differences were observed in serum LH, FSH, testosterone, or AMH concentrations in the present study. Experimental evidence on the reproductive endocrine effects of paroxetine in female rats remains limited and has yielded variable findings. El-Gaafarawi et al. (2005) reported that oral administration of paroxetine at 3.6 mg/kg/day for 10 days decreased serum LH and FSH levels while increasing testosterone levels in adult female rats [9]. In our previous study, paroxetine administered at 3.6 mg/kg once daily by oral gavage from postnatal day 21 for 10 weeks did not affect serum LH, FSH, or AMH levels in female rats [10]. In another study from our group, once-daily oral administration of paroxetine at 10 mg/kg/day for approximately 29 days significantly decreased serum AMH levels, whereas testosterone concentrations remained unchanged in adult female rats [8]. In the present study, a higher paroxetine dose of 20 mg/kg/day, administered via oral gavage for 8 weeks, did not produce significant changes in LH, FSH, testosterone, or AMH levels. In both of our previous studies, animals were sacrificed during diestrus, whereas El-Gaafarawi et al. (2005) did not report the estrous-cycle stage at the time of blood sampling [9]. Taken together, the available findings do not indicate a uniform pattern of reproductive endocrine responses to paroxetine in female rats. The variability among studies may, at least in part, reflect differences in developmental stage at treatment initiation, the dose and duration of paroxetine exposure, and blood-sampling conditions, including the estrous cycle stage at the time of sampling.

### 4.2. Vaginal Histological Findings

Our study showed that paroxetine significantly increased vaginal fibroblast counts, whereas irisin co-treatment significantly reduced fibroblast counts compared with the paroxetine group, suggesting that chronic paroxetine exposure was associated with increased fibroblast accumulation in vaginal tissue, whereas irisin co-treatment was accompanied by lower fibroblast counts.

Vaginal connective tissue undergoes continuous remodeling to preserve its structural integrity, elasticity, and supportive function. Fibroblasts, the dominant stromal cell population, play a central role in this dynamic process by sensing and responding to mechanical signals within the local microenvironment [54]. Consistent with this responsiveness, experimental evidence indicates that increased vaginal fibroblast numbers may accompany tissue remodeling-related changes, as such increases have been shown to accompany changes in collagen organization and increased collagen content in the anterior vaginal wall [55]. In a different experimental context, an experimental vaginal wound-healing model demonstrated that fibroblast proliferation was part of the local histological response and was accompanied by collagen deposition [56]. To our knowledge, the effects of paroxetine on vaginal fibroblasts have not been directly investigated. However, evidence from another tissue context suggests that paroxetine may influence fibroblast numbers. In a rat cutaneous wound-healing model, fibroblast counts were significantly higher in paroxetine-treated healthy rats than in saline-treated controls [57]. Although the tissue type and experimental setting differ substantially from those of the present study, this finding provides experimental evidence that paroxetine can modify fibroblast number under certain conditions and is therefore consistent with the increased vaginal fibroblast count observed following chronic paroxetine exposure in our study.

Similarly, direct evidence regarding the effects of irisin on vaginal fibroblasts remains scarce. Nevertheless, studies in fibroblasts from other tissues indicate that irisin can modulate fibroblast behavior in a context-dependent manner. In cardiac fibroblasts, higher irisin concentrations enhanced proliferation, whereas lower concentrations did not produce the same proliferative response [58]. More recently, irisin was also shown to increase the proliferation of human cardiac fibroblasts at specific concentrations under both normoglycemic and hyperglycemic conditions [59]. Similarly, irisin was shown to enhance the proliferation of human periodontal ligament fibroblasts across a range of concentrations and exposure periods [60]. Taken together, these findings indicate that the proliferative response of fibroblasts to irisin may vary depending on irisin concentration and experimental conditions. Against this background, the lower fibroblast count in the paroxetine + irisin group compared with the paroxetine group in the present study may reflect modulation of the paroxetine-associated increase in fibroblast number rather than a generalized inhibitory effect of irisin on fibroblasts.

In relation to plasma cell counts, significantly higher values were observed in the paroxetine, irisin, and paroxetine + irisin groups than in the sham control group. However, irisin co-treatment significantly reduced plasma cell counts compared with both the paroxetine and irisin groups. Plasma cells have been identified in the female reproductive tract, including the subepithelial layers of the vagina [61]. Moreover, vaginal plasma cell numbers have been shown to increase markedly following local immunization, indicating that this cell population is responsive to local immune stimulation [18]. To our knowledge, the direct effects of paroxetine on vaginal plasma cells have not been characterized. Plasma cells are terminally differentiated antibody-secreting cells of the B-cell lineage [62]. Therefore, studies examining B-cell and antibody-forming cell responses may provide indirect context for the potential effects of paroxetine on plasma cells. Oral paroxetine did not alter antigen-induced antibody-forming cell responses in mice [63]. In a different experimental and clinical context, however, three months of paroxetine treatment in patients with panic disorder was associated with a significant reduction in the proportion of circulating CD19^+^ B lymphocytes [64]. Taken together, these findings indicate variable effects of paroxetine across B-cell and antibody-forming cell-related immune endpoints, although their relevance to vaginal plasma cell abundance remains to be clarified. Similarly, direct evidence regarding the effects of irisin on vaginal plasma cells appears to be lacking. Nevertheless, indirect evidence is available from multiple myeloma, a malignancy characterized by the clonal proliferation of plasma cells within the bone marrow [65]. In this pathological context, irisin reduced myeloma cell viability and proliferation [65]. Although these findings were obtained in a malignant plasma-cell model and therefore do not directly reflect normal vaginal plasma cells, they provide evidence that irisin can influence cellular processes within the plasma-cell lineage. Against this background, the different plasma cell responses observed with irisin alone and in combination with paroxetine in the present study suggest that the effect of irisin on vaginal plasma cell number may depend on the treatment context rather than reflect a uniform stimulatory or inhibitory action.

Regarding TLR4 immunoreactivity, no significant differences were observed among the experimental groups. However, paroxetine significantly increased vaginal NF-κB immunoreactivity compared with the sham control group, whereas no other significant between-group differences were detected. As previously described, TLR4 plays an essential role in the recognition of pathogen-associated molecular patterns and in host defense against Gram-negative bacteria, transmitting signals mainly through MyD88- and TRIF-dependent pathways [21,22]. The MyD88-dependent pathway predominantly activates NF-κB, leading to the expression of inflammatory cytokine genes [22]. To our knowledge, the effects of paroxetine or irisin on TLR4 and NF-κB immunoreactivity in vaginal tissue have not been directly investigated. Although the effects of paroxetine on NF-κB have not been directly examined in female reproductive tissues, previous findings indicate that paroxetine can induce inflammatory alterations within these tissues. In our previous study, chronic paroxetine exposure significantly increased inflammatory cell infiltration in the endometrial stroma of female rats [10]. Although NF-κB was not assessed in that study, this finding provides a relevant inflammatory context for the increased vaginal NF-κB immunoreactivity observed following paroxetine exposure in the present study. Overall, these findings support an association between chronic paroxetine exposure and increased NF-κB immunoreactivity in vaginal tissue, with no significant alteration in TLR4 immunoreactivity. However, the increased NF-κB immunoreactivity should be interpreted as an indicator of altered inflammatory signaling rather than direct evidence of TLR4/NF-κB pathway activation.

Regarding PAS staining, severe (+++) apical reactivity was observed in the sham control group, weak (+) reactivity in the paroxetine group, and moderate (++) reactivity in both the irisin and paroxetine + irisin groups. The vaginal epithelium contains carbohydrate-rich components, including glycogen and glycoproteins, which are particularly associated with its superficial and apical regions [66,67]. PAS staining is widely used to detect polysaccharide-containing tissue components, including glycogen, glycoproteins, glycolipids, and mucins [68,69]. To our knowledge, no previous study has examined the effects of paroxetine or irisin on PAS reactivity in vaginal tissue. The weak apical PAS reactivity observed following paroxetine treatment may reflect a reduction in carbohydrate-associated PAS reactivity at the apical surface of the vaginal epithelium. Given the role of the vaginal epithelial surface in genital function, this histological finding may also have potential clinical relevance. Clinical evidence indicates that impaired vaginal lubrication may occur as a component of paroxetine-associated female sexual dysfunction; specifically, lubrication difficulties have been reported in women receiving paroxetine [70]. Nevertheless, because vaginal lubrication was not directly assessed, the functional significance of the reduced apical PAS reactivity in relation to lubrication remains uncertain. Thus, the reduced PAS reactivity is best interpreted as a local epithelial histological alteration associated with chronic paroxetine exposure. In contrast, the moderate PAS reactivity observed in the paroxetine + irisin group may suggest partial preservation of apical PAS reactivity compared with the paroxetine group. However, because moderate reactivity was also observed in the irisin group, the effect of irisin does not appear to represent a simple reversal of the paroxetine-associated change. Overall, these findings suggest that chronic paroxetine exposure was accompanied by a marked alteration in the apical PAS staining profile, whereas irisin may modulate this response in a treatment-dependent manner.

Taken together, the presence of vaginal histological and immunohistochemical alterations despite unchanged circulating reproductive hormone levels suggests that these local tissue responses may not be primarily driven by systemic endocrine changes. A local serotonergic mechanism represents one possible explanation. Serotonin (5-HT) has been demonstrated within the female reproductive tract of rats [71], and in an isolated rat uterus–vagina preparation, pelvic afferent fibers responded to chemical stimulation with 5-HT, indicating functional responsiveness of the female reproductive tract to serotonergic stimulation [72]. Although direct evidence linking 5-HT to vaginal fibroblast responses in rats is lacking, studies in fibroblasts from other rat tissues have shown that 5-HT can promote fibroblast proliferation and migration, through receptor-dependent mechanisms [73], providing a possible mechanistic context for the increased vaginal fibroblast count observed following paroxetine treatment. The increased plasma cell counts may likewise reflect alterations in local humoral immune activity within the vaginal mucosa, as plasma cells contribute to vaginal mucosal immunity and their numbers can increase markedly following local immune stimulation [18]. Serotonergic signaling may also provide a possible mechanistic context for the increased NF-κB immunoreactivity, as 5-HT1A-mediated signaling has been associated with increased nuclear translocation of NF-κB in activated lymphocytes [74]. Because NF-κB can be activated through multiple upstream signaling pathways and is not exclusively dependent on TLR4 [75], this may help explain the increased vaginal NF-κB immunoreactivity observed in the present study despite the absence of a significant change in TLR4 immunoreactivity. Overall, these observations provide a plausible mechanistic framework; however, because 5-HT concentrations and 5-HT receptor expression were not measured in vaginal tissue, the contribution of local serotonergic signaling to these tissue alterations remains to be clarified in future studies.

### 4.3. Limitations of the Study

Several limitations of the present study should be considered. First, the experiments were conducted in healthy female rats rather than in an experimental model of depression. Although this design allowed the effects of chronic paroxetine exposure to be examined without disease-related confounding factors, the findings may not fully reflect responses occurring in the presence of an underlying depressive condition. Second, the control procedures were not fully matched across all experimental groups. The irisin-only group did not receive daily vehicle gavage, whereas the paroxetine-only group did not undergo anesthesia, sham surgery, or vehicle-pump implantation. Accordingly, the potential contributions of these procedural differences to behavioral, physiological, and inflammatory outcomes cannot be entirely ruled out and should be considered when interpreting the findings. In addition, the same male and female stimulus animals were used throughout behavioral testing, their positions were kept constant rather than randomized or counterbalanced, and the order of testing across treatment groups was not randomized. Therefore, potential stimulus-specific, position-related, or testing-order effects on approach and investigation behavior cannot be excluded. Third, the a priori sample size calculation was not based on a single prespecified primary outcome and should therefore be regarded as an overall design-level estimate rather than an outcome-specific power determination. In addition, the study was conducted as a single experimental cohort and was not independently replicated in a separate cohort. No experiment-wide adjustment for multiplicity was applied across the multiple outcomes examined. Accordingly, the analyses were exploratory in nature, and the increased risk of type I error should be considered when interpreting findings close to the *p* = 0.05 threshold. Fourth, the study focused on histological and immunohistochemical alterations in vaginal tissue and did not include direct functional assessments of vaginal physiology, such as lubrication or tissue elasticity. Furthermore, paroxetine concentrations were not measured in vaginal tissue; therefore, the extent to which local drug exposure may have contributed to the observed tissue alterations remains unknown. Regarding the immunohistochemical analyses, TLR4 and NF-κB staining followed our previously published protocol; however, positive and negative controls were not included in the present study, a limitation that should be considered when interpreting these findings. Finally, irisin was evaluated at a single dose of 100 ng/kg/day, selected based on its previously demonstrated biological activity in female rats. The present study was not designed to examine dose–response relationships; therefore, whether different irisin doses would produce similar or distinct effects remains to be determined. Future studies incorporating depression models, more closely matched procedural controls, functional vaginal measurements, local drug quantification, and evaluation of different irisin doses would help further define the biological significance and mechanisms of these findings. From a translational perspective, the vaginal tissue alterations observed here may warrant investigation in relation to clinical symptoms such as impaired lubrication or altered tissue compliance. However, these functional consequences cannot be inferred from the present histological findings alone, and whether therapeutic interventions could modify these responses requires direct evaluation in appropriate translational and clinical studies.

## 5. Conclusions

In conclusion, chronic paroxetine exposure was associated with a distinct pattern of reproductive effects characterized by behavioral and local vaginal tissue alterations in the absence of significant changes in circulating reproductive hormones. The dissociation between the preserved endocrine profile and the changes observed in vaginal stromal, immune-inflammatory, and epithelial parameters including altered apical PAS reactivity, suggests that the reproductive changes observed following prolonged paroxetine treatment may involve local tissue-level responses that are not necessarily reflected by systemic hormonal measurements. Irisin influenced several of these vaginal responses, but its actions were not consistent across the evaluated outcomes and did not extend to the paroxetine-associated behavioral changes. Moreover, the effects observed with irisin alone indicate that its actions cannot be interpreted solely as modifications of paroxetine-associated changes. Collectively, these findings demonstrate that chronic paroxetine exposure is associated with local vaginal tissue alterations in addition to behavioral changes, while the effects of irisin vary across the evaluated outcomes. Further studies integrating local molecular signaling with functional vaginal outcomes are needed to determine the significance of these tissue alterations.

## Figures and Tables

**Table 1:** Group-specific treatment and procedural control schedule for the four experimental groups.

Group	Weeks 1–8: Daily Oral Gavage	28-Day Mini-Osmotic Pump Infusion
Sham control	Deionized water	Vehicle-filled pump
Paroxetine	Paroxetine, 20 mg/kg/day	No pump implantation
Irisin	No gavage	Irisin, 100 ng/kg/day
Paroxetine + irisin	Paroxetine, 20 mg/kg/day	Irisin, 100 ng/kg/day

**Table 2:** Vaginal epithelial PAS reactivity in rats treated with sham control, paroxetine, and/or irisin. *n* = 10 rats per group. (+): weak; (++): moderate; (+++): severe.

Parameters	Sham Control	Paroxetine	Irisin	Paroxetine + Irisin
PAS Reaction	+++	+	++	++

## Data Availability

All data generated and/or analyzed in the present study are included in this paper and can be obtained from the corresponding author upon reasonable request.
